# Type B Lactic Acidosis: A Very Rare but Fatal Complication of Gastrointestinal Solid Tumor

**DOI:** 10.7759/cureus.56788

**Published:** 2024-03-23

**Authors:** Fuad I Abaleka, Genanew Bedanie, Diego Olavarria Bernal, Selome F Yewedalsew, Tasur Seen

**Affiliations:** 1 Internal Medicine, Covenant Medical Center, Lubbock, USA; 2 Internal Medicine, Texas Tech University Health Sciences Center, Lubbock, USA; 3 Internal Medicine, State University of New York (SUNY) Downstate Health Science, New York, USA; 4 Gastroenterology, Icahn School of Medicine at Mount Sinai, Elmhurst Hospital, Elmhurst, USA

**Keywords:** malignant solid tumor, colon cancer liver metastasis, anion gap mmetabolic acidosis, type b lactic acidosis, colorectal cancer

## Abstract

Type B lactic acidosis, secondary to solid cancer, is very rare. It is mostly seen in patients with hematological malignancies. Although its exact pathogenesis is unknown, it is believed to be caused by overproduction and the inability of tumor cells to remove lactate. In the last 26 years, a systematic review of the literature only identified two previous reports of colorectal cancer-related type B lactic acidosis. Here, we report the third case of severe type B lactic acidosis due to stage IV colorectal with liver metastasis. Besides, this case is unique in that serum lactate levels reaching as high as 24 mmol/L were not reported in association with colorectal cancer. In most cases, the prognosis is still very poor because there are no standardized treatment recommendations. Early chemotherapy is still the only intervention that provides some survival benefits.

## Introduction

Lactic acidosis frequently occurs in critically ill patients and is the most common cause of metabolic acidosis in hospitalized patients. Severe lactic acidosis is defined as a blood lactate level greater than 4.0 mmol/L (normal range: 0.7 to 2.7 mmol/L). Type A lactic acidosis is seen in cases of reduced soft tissue perfusion, such as heart failure, sepsis, and cardiopulmonary arrest. Type D lactic acidosis may occur in patients with gastrointestinal malabsorption and short bowel syndrome, and the cause is believed to be intestinal bacterial overgrowth resulting in excessive transport of carbohydrates to the small intestine and excessive production and absorption of D-lactate [1]. Type B lactic acidosis is rare and is characterized by the absence of systemic hypoperfusion. It is thought to be caused by disruption of cellular metabolism, but the cause is still unclear as many theories are described in the literature. It can be seen in malignancy, diabetic ketoacidosis, alcohol consumption, use of certain medications (e.g., metformin, [2] propofol), and HIV antiretroviral treatment [3]. Colon cancer is the fourth-leading cause of cancer in the United States, with approximately 152,810 new cases each year [4]. The liver remains the most common metastatic site of colon cancer. Here, we present a rare case of metastatic colorectal cancer that developed type B lactic acidosis and had a poor outcome. This article was previously presented as a poster at the 2020 American College of Gastroenterology Virtual Meeting on October 25, 2020 [5]. 

## Case presentation

A 61-year-old man with a past medical history of type-2 diabetes and hypertension was admitted due to fatigue, nausea, shortness of breath, and abdominal pain of a few days’ duration. He reported intermittent watery diarrhea for two weeks, followed by worsening fatigue, lower abdominal pain, and shortness of breath for 2-3 days. The patient was recently diagnosed with stage IV invasive colon cancer of the rectosigmoid area with liver metastasis three weeks ago and was about to begin chemotherapy when these symptoms appeared. The patient apparently was not compliant with his medications; he was not taking his home medications (Jardiance 10mg ) for his diabetes and (Lisinopril 10mg ) for hypertension. 

Initial vital signs: blood pressure of 133/70 mmHg, heart rate of 115 beats per minute, respiratory rate of 25 breaths per minute, temperature of 97.8 F, and oxygen saturation of 97% in room air.

On initial examination, he was alert and oriented (x4) and in mild distress. He was emaciated, and a Chemo port was noted. Has a dry mouth and tongue and mild icteric sclera. No lymphadenopathy. Chest examination with good air entry, no added sound bilaterally. Cardiovascular exam with flat JVD, S1, and S2 heard, no murmur or gallop. The abdomen is full, and the liver is easily palpable with an irregular surface and firm consistency. Right upper quadrant deep palpation reveals mild direct tenderness without guarding or rigidity. Otherwise, normoactive bowel sounds.

Initial workup revealed a white blood cell count of 12.6K, a hemoglobin of 13.6 gm/dL, and a platelet of 346K. Alanine transferase 74 units/L, aspartate transaminases 111 units/L, alkaline phosphatase 724 units/L, total bilirubin 2.0mg/dl, albumin 3.5 gm/dl, INR 1.23. Arterial blood PH 7.2, bicarbonate 5.9, lactic acid 13.7 mmol/L, glucose 123 mmol/dl, sodium 136 mmol/L, potassium 4.3 mmol/L, creatinine 0.9 mg/dl, BUN 21 mg/dl, anion gap without potassium 31, acetone was negative, and urine analysis was unremarkable. The chest X-ray was negative for acute pathology.

**Figure 1:**
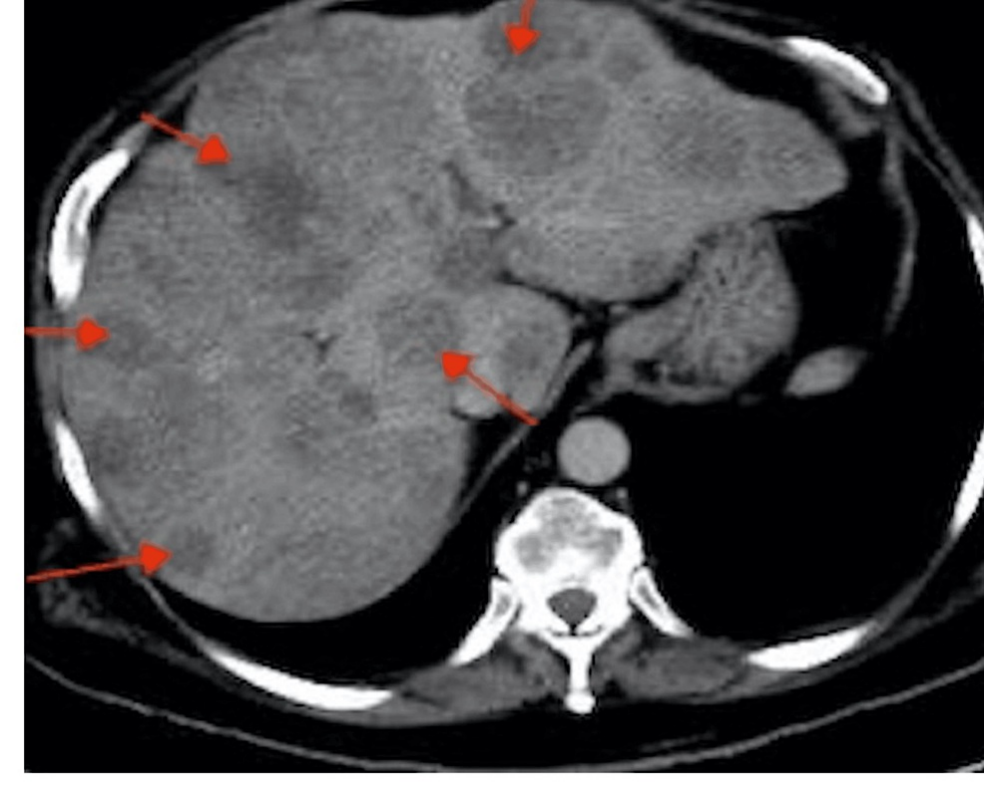
Abdominal computed tomography shows multiple hypoattenuating lesions (red arrows) in the liver, indicating metastatic disease.

**Figure 2:**
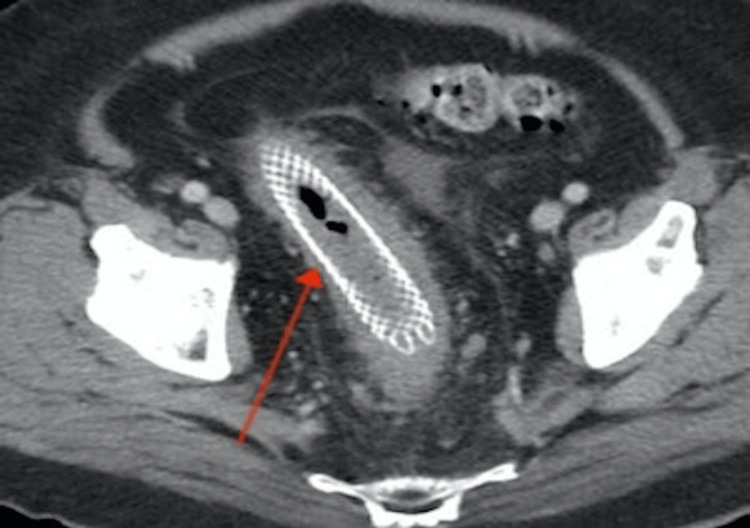
Abdominal and pelvic computed tomography revealed a moderate-sized pericolonic mass in the pelvis with enlarging satellite nodules. Rectosigmoid Interval stent placement seen.

Abdomen/pelvis computed tomography revealed a rectosigmoid mass and liver metastases (Figures 1, 2) with no perforation or any other cause of acute pathologies. The patient was admitted to the ICU, and he was started initially on the sepsis protocol with IV fluid (an initial bolus of 0.9% saline), broad-spectrum IV antibiotics with vancomycin and Zosyn for coverage of sepsis from unknown sources, and a bicarb drip. A surgical evaluation ruled out an acute abdomen. On day three, the patient was not febrile, his blood pressure was stable, his culture remained negative, but his lactic acidosis continued to worsen despite aggressive IV fluid, an IV bicarb drip, and antibiotics. Hence, sepsis as a cause of lactic acidosis in this particular case was ruled out. Type D lactic acidosis is also unlikely in this patient, as he had no risk factors, such as a history of short bowel syndrome or exposure to propylene glycol. The possibility of type B lactic acidosis was entertained, given his recent diagnosis of colon cancer with extensive liver metastasis, and he was started on chemotherapy with the FOLFOX regimen along with IV thiamine. The patient was closely monitored in the ICU, but his condition continues to decline. Serial blood tests for serum lactate and anion gap without potassium continued to increase, but serum PH improved (Table 1). On day eight, after admission, the patient became more confused, abdominal pain became constant, and the family decided to proceed with comfort care measures. After about 12 hours, the patient expired. 

**Table 1:** shows serum lactate level and anion gap without potassium worsening but blood PH level and serum carbon dioxide improving with bicarb drip.

	Hospital Day					
	1	2	3	4	5	6
Serum lactate (normal = 0.5-2.2 mmol/L)	13.7	16.3	15	17.5	20	24
Serum carbon dioxide (normal = 21-29 mmol/L)	7	9.3	10	7	15	20
Blood pH (normal = 7.35-7.45)	7.21	7.31	7.34	7.36	7.40	7.37
Anion Gap w/o potassium (normal = 4-12 mmol/L)	31	36	32	39	35	39

## Discussion

Our patient is 61 years old and has type B lactic acidosis due to stage IV colon cancer with extensive liver metastatic disease. Type B lactic acidosis may be associated with malignancy, whose pathophysiology is unclear, and various hypotheses have been proposed in the literature.

The liver plays an important role in lactate metabolism: 80% of lactate is metabolized to glucose by gluconeogenesis in the liver, and the remainder is metabolized in the kidneys. More than 90% of solid cancers associated with type B lactic acidosis show liver metastatic disease [6]. In contrast, there are several types of hematological malignancies that present with this problem with little or no hepatic involvement [7]. Liver is the most common site of metastasis in colon cancer, with approximately 20% of newly diagnosed cases of metastases [8]. Although many patients develop metastases to the liver, the incidence of type B lactic acidosis is still low, indicating multifactorial causes of this problem.

The Warburg effect has been described, in which tumor cells change their metabolism to the lactate pathway, resulting in increased intracellular NADPH, redirection of glucose into the pentose phosphate shunt, and increased biosynthesis of nucleosides and amino acids. Tumor cells replicate even in hypoxic tumor environments [9].

Thiamine is a cofactor for pyruvate dehydrogenase and is required for the conversion of pyruvate to acetyl-CoA and subsequent participation in the Krebs cycle. In thiamine deficiency, excess pyruvate is converted to lactic acid by the lactate dehydrogenase hormone, causing lactic acidosis. This may occur in patients using total parenteral nutrition. In this condition, thiamine supplementation may decrease lactic acid levels [10].

Tumor necrosis factor-α, an inflammatory cytokine secreted by hematological malignancies, is believed to inhibit pyruvate dehydrogenase and increase lactate production. However, its action in solid tumors is unclear. Even though few cancers overexpress hexokinase- and insulin-like growth factors. This can increase glycolysis and pyruvate production [11].

It is believed that tumors with a very rapid proliferation index grow beyond their blood supply, and increased expression of glycolytic enzymes increases glycolysis and anaerobic metabolism in a relatively hypoxic tumor microenvironment, resulting in ischemia and necrosis; thus, tumor angiogenesis plays an important role in this condition. In the presence of diffuse liver metastases, lactate production exceeds the liver’s elimination capacity, resulting in severe metabolic acidosis. In our particular case of advanced colon cancer with extensive liver metastasis, lactic acidosis is thought to be due to increased lactic acid production caused by disruption of the cellular metabolism of tumor cells and decreased lactic acid clearance by the liver due to extensive liver involvement. Our case presented with severe lactic acidosis. His serum lactic acid was initially 14 mmol/L but trended up to 24 mmol/L despite aggressive fluid, bicarb drip, and antibiotic therapy in the ICU. According to a literature review, a serum lactate level of 24 mmol/L is the highest serum lactate level reported in a solid tumor. Among solid tumors, only one case of serum lactate levels up to 24 mmol/L in a patient with metastatic small cell cancer of the lung has been reported so far [12]. Our case is unique in that serum lactate levels reaching 24 mmol/L were not reported in association with colorectal cancer.

Most common cases of type B lactic acidosis are seen with hematological malignancies. Numerous cases are reported with multiple myeloma [13], lymphoma, and leukemia [14]. Reports on solid cancer are limited. In a review of the literature over the past 26 years, there were only two case reports of type B lactic acidosis due to colon cancer [6]. Our case is the third report of type B lactic acidosis in association with colorectal cancer, but the first report of a case with a very high serum lactate level (24 mmol/L) due to colorectal cancer from 1998 to 2024 [6].

## Conclusions

Type B lactic acidosis is a rare but fatal complication of malignancy. It usually appears in association with hematologic malignancy but is very rare with colorectal tumors. When a patient with advanced cancer presents with severe lactic acidosis, it is appropriate to first rule out common causes of lactic acidosis, such as sepsis, hypoxia, tissue ischemia, or hypoperfusion and initiate treatment immediately. All metastatic cancer patients with extensive liver disease and unexplained high anion gap metabolic acidosis (high anion gap, high serum lactate levels, and no other causes of lactic acidosis) should be considered to have this fatal complication. Currently, there is no standard of care or therapy available. A bicarbonate infusion improves blood PH but has no survival benefit. If possible, early chemotherapy targeting the primary tumor should be considered because this is the only intervention that provides some benefit in terms of survival.
